# Clinical Re-Audit of the Use of Melatonin for Patients Within CAMHS

**DOI:** 10.1192/bjo.2026.11746

**Published:** 2026-06-30

**Authors:** Alexandra Babeanu, Mert Kuzu, Ana Gasparyan

**Affiliations:** Tees, Esk and Wear Valleys NHS Foundation Trust (TEWV), York, United Kingdom

## Abstract

**Aims:**

Melatonin is an endogenous hormone used to manage insomnia in children with neurodevelopmental disorders. Within the Tees, Esk & Wear Valleys NHS Foundation Trust, the Shared Care Guideline (PHARM-0025-V5) mandates specific standards for prescribing and documentation. Following an initial audit in March 2024 that identified partial compliance, this re-audit aimed to assess progress in the York CAMHS ADHD team’s adherence to these guidelines.

**Methods:**

A retrospective clinical re-audit was conducted between October 9th and 12th 2025. Data were collected from 50 electronic patient records (CITO) for children diagnosed with ADHD who were commenced on Melatonin between March 2024 and October 2025. The audit tool assessed five key criteria: documentation of prescribing rationale, provision of oral/written information, advice on short-term use/treatment breaks, and adherence to correct licensed formulations (Adaflex/Slenyto).

**Results:**

Significant improvement was noted in prescribing correct formulations, rising from 70% in the first cycle to 90% (45/50). Documentation of the rationale for formulation switches also improved to 50% (2/4 applicable cases). However, compliance remained low in other areas:

Prescribing Rationale: Stable at 76% (38/50) compared to 77% in the first cycle.

Information Provision: Recorded evidence of oral/written information declined from 33% to 18% (9/50).

Short-term Use Advice: Remained low at 8% (4/50) compared to 10% in the previous cycle.

**Conclusion:**

The re-audit demonstrates that targeted feedback effectively improved technical prescribing adherence regarding medication choice. However, a significant “documentation gap” persists concerning patient-centered communication and review planning. Proposed actions include delivering team-teaching sessions to emphasize the necessity of documenting patient discussions and implementing standard phrases in electronic notes to ensure comprehensive record-keeping.

